# New Technique: A Novel Femoral Derotation Osteotomy for Malrotation following Intramedullary Nailing

**DOI:** 10.1155/2012/837325

**Published:** 2012-11-20

**Authors:** S. Jagernauth, A. J. Tindall, S. Kohli, P. Allen

**Affiliations:** ^1^Trauma and Orthopaedic Department, Royal London Hospital, North East Thames Rotation, London E1 1BB, UK; ^2^Queen Elizabeth II Hospital, Woolwich, UK; ^3^Princess Royal University Hospital, Bromley, UK

## Abstract

A 19-year-old female patient sustained a closed spiral midshaft femoral fracture and subsequently underwent femoral intramedullary nail insertion. At followup she complained of difficulty in walking and was found to have a unilateral in-toeing gait. CT imaging revealed 30 degrees of internal rotation at the fracture site, which had healed. A circumferential osteotomy was performed distal to the united fracture site using a Gigli saw with the intramedullary femoral nail in situ. The static distal interlocking screws were removed and the malrotation was corrected. Two further static distal interlocking screws were inserted to secure the intramedullary nail in position. The osteotomy went on to union and her symptoms of pain, walking difficulty, and in-toeing resolved. Our paper is the first to describe a technique for derotation osteotomy following intramedullary malreduction that leaves the intramedullary nail in situ.

## 1. Case Report

A 19-year-old female sustained a closed spiral midshaft fracture of her right femur following a road traffic accident. The fracture was treated using a femoral intramedullary nail with one dynamic proximal screw and two lateral static distal locking screws. There were no intraoperative complications noted.

**Figure 1:**
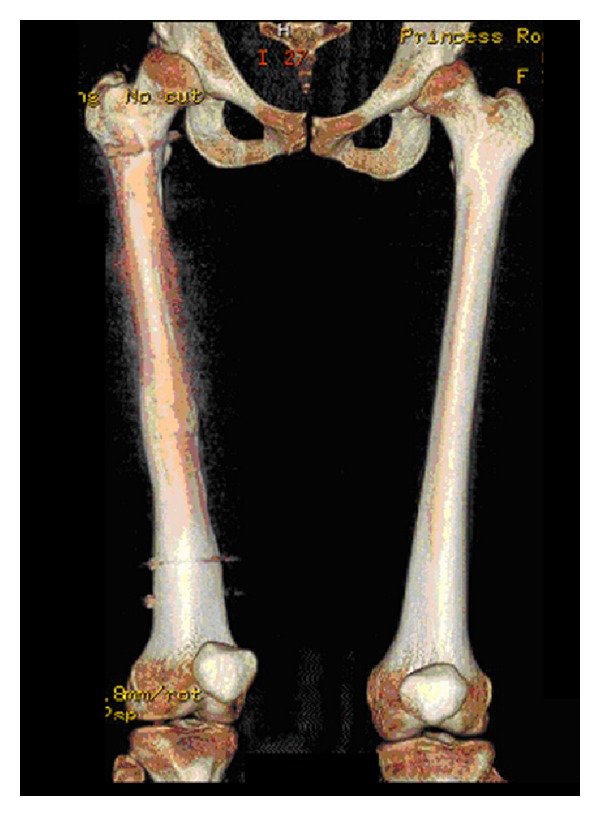
CT reconstruction showing rotational deformity of the right femur.

After six months, she complained of difficulty walking. On examination she was noted to have an internally rotated right leg with her patella pointing medially. She also walked with a painful in-toeing gait on the right. No leg length discrepancy was found. Her wounds had healed well and she complained of no rest pain. Radiographs of her femur showed signs of union of the fracture. A CT scan of her right femur revealed approximately 30 degrees of internal rotation distal to the fracture site (see Figure 1).

A decision was made to proceed with a derotational osteotomy, leaving the femoral nail in situ. At operation a lateral approach to the femur was performed. One K wire was inserted into the femur proximal to the fracture site and another inserted at 30 degrees of internal rotation distal to the fracture site. This angle was measured using a protractor normally used for the Maquet tibial osteotomy. A circumferential femoral osteotomy was made around the femoral intramedullary nail using a Gigli saw approximately 5 cm proximal to the distal interlocking screws. The bone was held above and below the osteotomy site using bone clamps. The lateral static distal interlocking screws were then removed and the malrotation was corrected. This brought the guidewires parallel, thereby correcting the malrotation deformity. Two antero-posterior static distal interlocking screws were used to secure the intramedullary nail and freeze-dried bone graft was inserted around the osteotomy site. 

**Figure 2:**
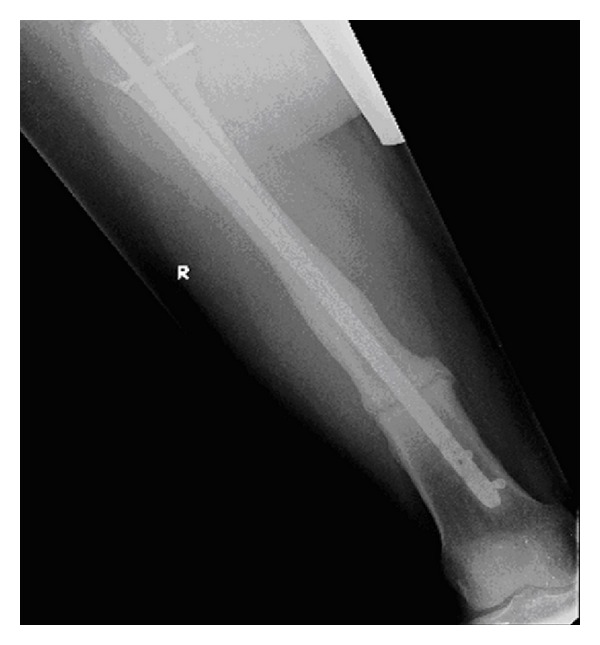
AP radiograph following corrective osteotomy of the right femur.

 The patient went on to make a good postoperative recovery and at her six-month followup she had no pain and walked with a normal gait. The osteotomy site had united both clinically and radiologically (see Figure 2) and left the patient with no residual symptoms.

## 2. Discussion

Minor rotational malalignments are common following intramedullary nailing of closed femoral shaft fractures but gross rotational malalignments are rare. One study revealed 28% of patients had a rotational malalignment of 15 degrees or more after intramedullary nailing for femoral fractures [1]. Although the hip can correct some rotatory malunion to a degree, the new *range* of motion of the hip joint (as opposed to its unchanged *arc* of motion) can cause difficulties with more demanding activities such as running, sports, and climbing stairs.

Many methods have been described to correct symptomatic rotational deformity, either using external fixation [2], internal plating [3], or intramedullary devices [4]. The results of such corrections tend to be good with symptoms resolving in the majority of cases and major complications being rare. 

Previously described methods rely on adding in a new fixation device. This usually necessitates removal of the original device which can be technically difficult, leaves potential stress risers, and takes extra time. More importantly, replacement of an intraosseous device with an extraosseous one will strip the last source of blood supply to the bone. Using an external circular frame avoids this problem, but introduces new concerns of infection and can be difficult for the patient. Both methods have the potential to introduce new malalignments in either the coronal or sagittal plane. 

Our method introduces the option to perform a derotational osteotomy using the existing fixation device. It allows for stability in the coronal and sagittal planes during the rotational correction and helps the surgeon “dial in” the correct correction. It does not violate any blood supply to the femur and is minimally invasive. There has been much discussion regarding whether to use larger reamed nails at the expense of potential damage to the blood supply [5]. Our method has the advantage of using a large nail for stability and fatigue strength with none of the potential problems associated with further reaming.
